# Advances in Sphingolipidoses: CRISPR-Cas9 Editing as an Option for Modelling and Therapy

**DOI:** 10.3390/ijms20235897

**Published:** 2019-11-24

**Authors:** Renato Santos, Olga Amaral

**Affiliations:** Human Genetics Department, Unit of Research and Development, Nacional Health Institute Doctor Ricardo Jorge, 4000-055 Porto, Portugal; rnt.ad.santos@gmail.com

**Keywords:** sphingolipidoses, lysosomal storage diseases, CRISPR-Cas9, Gaucher disease, Fabry disease, Tay–Sachs disease, Sandhoff disease, Niemann–Pick disease, Krabbe disease, GM1-gangliosidosis, gene editing

## Abstract

Sphingolipidoses are inherited genetic diseases characterized by the accumulation of glycosphingolipids. Sphingolipidoses (SP), which usually involve the loss of sphingolipid hydrolase function, are of lysosomal origin, and represent an important group of rare diseases among lysosomal storage disorders. Initial treatments consisted of enzyme replacement therapy, but, in recent decades, various therapeutic approaches have been developed. However, these commonly used treatments for SP fail to be fully effective and do not penetrate the blood–brain barrier. New approaches, such as genome editing, have great potential for both the treatment and study of sphingolipidoses. Here, we review the most recent advances in the treatment and modelling of SP through the application of CRISPR-Cas9 genome editing. CRISPR-Cas9 is currently the most widely used method for genome editing. This technique is versatile; it can be used for altering the regulation of genes involved in sphingolipid degradation and synthesis pathways, interrogating gene function, generating knock out models, or knocking in mutations. CRISPR-Cas9 genome editing is being used as an approach to disease treatment, but more frequently it is utilized to create models of disease. New CRISPR-Cas9-based tools of gene editing with diminished off-targeting effects are evolving and seem to be more promising for the correction of individual mutations. Emerging Prime results and CRISPR-Cas9 difficulties are also discussed.

## 1. Lysosomal Storage Diseases and Sphingolipidoses

The present study was based on high-quality, peer-reviewed publications available at NCBI Pubmed until October 2019. The following work aims to provide a review covering the application of CRISPR-Cas9, with a focus on sphingolipidoses.

Lysosomal storage diseases (LSDs) are an important subdivision of inherited metabolic diseases. As a group, these monogenic diseases are estimated to affect 1 in 7500 newborns [1].

Generally, LSDs are multisystemic and degenerative. Patients present a continuum of disease severity, which are usually classified by the type of disorder and the age of onset of clinical signs. Different types of defects may underlie LSDs, including, but not limited to, defects in lysosomal hydrolases, lysosomal membrane proteins, activator proteins, or transport proteins. Nevertheless, they all present a common feature, which is the storage of macromolecules in the lysosomes. When one of the several lysosomal hydrolases is dysfunctional, or has lost activity, due to mutations or incorrect protein folding, the related substrate accumulates inside the cell. Depending on the type of storage materials, LSDs are classically divided in subgroups that reflect the analogous affected lysosomal pathway. Such is the case, for example, of the sphingolipidoses, mucopolysaccharidoses, and glycoproteinoses. Additionally, there are diseases involving integral membrane proteins, disorders of lysosome-related organelles, and disorders involving lipofuscin [2].

**Figure 1:**
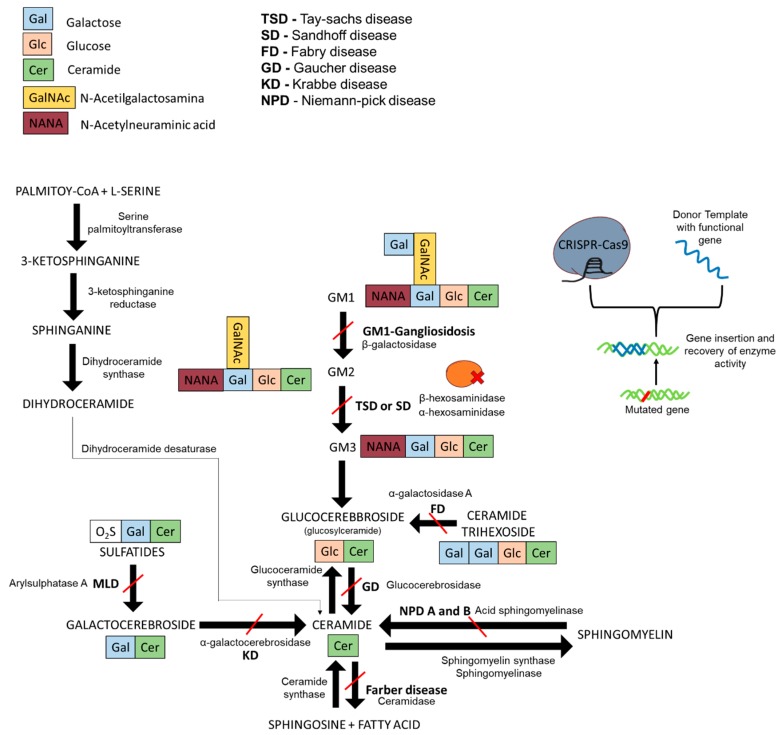
GM1-gangliosidosis degradation pathway, sphingolipid synthesis pathway, and enzyme activity recovery with CRISPR-Cas9 and DNA template. The red lines describe the interrupted pathway of degradation due to a specific dysfunctional enzyme. In bold are the names of the sphingolipidoses caused by the mutated enzymes and the consequent interrupted pathways. Scheme drawn on the basis of the bibliography used in this review.

Sphingolipidoses are a subgroup of LSDs usually caused by defective sphingolipid hydrolases that act upon the sphingolipid degradative pathway. Most sphingolipidoses present autosomal recessive inheritance. In some population groups, a few mutations account for a large proportion of the mutant variants. Nevertheless, a myriad of mutations can cause these disorders. Mutated genes coding for sphingolipid hydrolases or deregulation of lysosomal-related proteins are at the basis of sphingolipidoses. In these diseases, cells are unable to degrade specific lipids (Figure 1), which then accumulate inside the cell until reaching toxic or nonfunctional levels, leading to multisystemic pathologies [3,4,5].

These rare disorders have been associated with other frequent diseases, most likely because the toxic effects on the cells makes them prone to the development of other, more common diseases [6,7]. For instance, causal mutations of Gaucher disease are considered to constitute a risk for Parkinson’s disease. Therefore, diseases involving mutant glycosphingolipids represent a significant multidimensional problem. Moreover, recent results in Niemann–Pick A/B demonstrate a tight link between cellular sphingolipid metabolism and immunity, which supports the hypothesis of a central role of sphingolipids in processes beyond those of the lysosome [8]. Recently, a transcription factor EB (TFEB), which is responsible for the transcription coordination of most lysosomal genes, has been identified, this transcription controller provides a new tool to manipulate and study lysosomal diseases [9]. The discovery of TFEB led to the search for a motif associated with genes with lysosomal function, and the coordinated expression resulted in the identification of a new mechanism, the coordinated lysosomal expression and regulation (CLEAR) [10].

Although the LSDs are rare, research in the field has been abundant; LSDs have been the stage for the development of new therapies and have provided knowledge that extends beyond LSDs.

### 1.1. Gaucher Disease

The most common sphingolipidosis is Gaucher disease (GD). It is caused by mutations in the *GBA1* gene that encodes lysosomal acid-β-glucocerebrosidase (GCase), the enzyme responsible for the degradation of glucosylceramide (GlcCer). The Gaucher′s macrophage is the classic pathologic hallmark of the disease, presenting a lysosomal compartment enlarged by the storage of undegraded glycolipid molecules. These cells have a pivotal role in the development of the disease since they are capable of inducing local tissue reactions [1].

Depending on the level severity, deterioration, and type of impairment, GD can be categorized in three main groups, type I, II, and III. The most frequent form of GD is type I, also known as nonneuropathic GD as it spares the nervous system. Thus, it is considered to be the less aggressive form of GD. It can be subdivided according to the age of onset and may have clinical onset at any time during the human life cycle [6,11]. Types II and III are characterized by the presence of primary neurologic disease affecting the central nervous system (CNS). Type II is the most severe form of GD—it has severe and rapid degeneration leading to death before 2–4 years of age [12,13]. Type III usually has a later onset with slower progression. In spite of the three conventional GD classifications, GD presents an important continuum of clinical forms. The symptoms of Gaucher are extensive, affecting many organs systems, such as skeletal, cardiovascular, and neurological. In some cases, GD variants have been associated with other neurological diseases such as Parkinson’s disease and peripheral neuropathy [6,11].

GD can be caused by *GBA1* mutations or, less frequently, by mutations in the gene coding for the activator protein saposin C. The application of new sequencing approaches leads to a continuously growing number of *GBA1* mutations. Nevertheless some mutations have been reported as more frequent in specific population groups [12,13,14].

The degree of endoplasmic reticulum retention (ER) and proteasomal degradation has recently been proposed as an important factor influencing the outcome of GCase mutants. Different mutations in *GBA1*, which impact the ability of the protein to fold correctly, present variable levels of ER retention and undergo ER-associated degradation (ERAD) in the proteasome [15].

It is known that GD patients are at a higher risk to develop Parkinson′s disease (PD) in comparison to the non-GD population. Thus, the mutated GCase seems to be a predisposing factor for the development of PD. Mutant GCase contributes to the accumulation and aggregation of α-synuclein, one of the main causes of PD, which may be a reason for pathological interaction [16]. The presence of mutant GCase is also thought to lead to the enhancement of neuronal vulnerability to neurodegenerative processes, triggered by increased alpha-synuclein expression [17] and by ERAD, which is also known to be involved in the process leading to Parkinson′s disease [15].

### 1.2. Fabry Disease

Fabry disease (FD) is a sphingolipidosis caused by a mutated alpha-galactosidase A, encoded by the *GLA* gene. FD follows an X-inheritance pattern, but methylation skewing can also lead to female presentation of the disease. When the enzyme is nonfunctional or not present, its substrate, globotriaosylceramide (Gb3), accumulates in the lysosomes [18,19].

The classic form of FD is clinically heterogeneous, with different symptoms along the patient’s life, and usually has more severe clinical expression in males than in females. The lifespan is decreased, often due to renal failure, heart diseases, and strokes. However, there are also reports of late-onset Fabry disease in males, involving only a single organ system (cardiac or renal) due to missense mutations that lead to sufficient residual enzyme activity that prevent symptoms in childhood and early adulthood [20,21].

### 1.3. GM2-Gangliosidoses

Tay–Sachs Disease (TSD) is one of the most common GM2-gangliosidoses, the substrate is the main glycolipid of neuronal cell plasma membranes. GM2-gangliosidoses are a group of autosomal recessive LSDs related to deficiency in lysosomal hexosaminidases (Hex). Deficient enzyme activity leads to abnormal accumulation of GM2 ganglioside and impaired neuronal cellular activity [22]. Defects in the gene-coding ganglioside GM2 activator protein (*GM2A*) also lead to TSD. The Hex enzymes are all dimers encoded by the same two genes, *HEXA* (encodes α subunit) and *HEXB* (encodes β subunit). HexA enzyme is composed of subunits α and β, and HexB by two β subunits [23]. In the case of TSD, HexA enzyme is mutated whereas in Sandhoff disease (SD), it is HexB that is mutated.

SD is classified in three main age-related forms: (1) infantile, with progressive neurologic impairment, hypotonia, bilateral cherry-red spots in the retina, and seizures; (2) juvenile, with dementia, cerebellar ataxia, mental retardation, and spinal muscular atrophy; and (3) adult, with spinocerebellar degeneration or motor neuron disorders [24].

TSD is also classified by age of onset. The infantile form is characterized by mental and motor developmental delay, hypotension, cherry-red spots, and dysphagia; the juvenile form is characterized by ataxia and spasm progression; and the later-onset form is characterized by gradual decline in motor, cerebral, and spinocerebellar function [25].

Variant B1, particularly prevalent in populations of Northern Iberian descent, particularly in individuals of Portuguese ancestry, is due to a higher frequency of a specific HEXA mutation. This atypical juvenile variant is characterized by differential activity towards sulphated and nonsulphated substrates. The deficiency in this enzyme has altered kinetic properties due to active site disturbance. In most cases, this variant is caused by at least one c.533 G > A allele [26,27].

### 1.4. Niemann–Pick Disease

Niemann–Pick diseases (NPD) are autosomal recessive inherited diseases subdivided into hydrolase deficient entities or trafficking-impaired disorders. In acid sphingomyelinase-deficient NPD (ASM-deficient NPD), underlying defects are due to *SMPD1* gene mutations (type A, B, and intermediate forms), whereas in NP type C (NPC), which is currently described as a cholesterol trafficking defect, is due to *NPC1* or *NPC2* gene mutations [28,29]. NPC was historically classified as a sphingolipidosis, but it is not caused by a deficiency in sphingolipid catabolism. Rather, it is caused by a cholesterol trafficking defect.

The ASM is a lysosomal enzyme which degrades sphingomyelin. When the enzyme is not present or is nonfunctional, sphingomyelin accumulates in different organs. Type A NPD leads to hepatosplenomegaly and severe CNS problems in infancy, with a frequent lifespan of only 2–3 years after birth. Type B NPD causes hepatosplenomegaly and lung problems, but typically does not impact the CNS. Frequently, NPD diseases cause lipid storage and foam cell infiltration in tissues, pulmonary insufficiency, and central nervous system issues [30,31].

### 1.5. Krabbe Disease

Like most LSDs, Krabbe Disease (KD) is an autosomal recessive disease. Its underlying causal deficiency is in the lysosomal enzyme β-galactocerebrosidase (Galc), which results from a mutated *GALC* gene that encodes that enzyme. When deficiency of such enzyme is present, galactosylceramide, the major natural substrate, accumulates in lysosomes. This sphingolipid contains sphingosine, galactose, and fatty acid, and is almost exclusively localized in the myelin sheath [32]. Besides galactosylceramide, Galc has other substrates: galactosylsphingosine or psychosine, monogalactosyldiglyceride, and the precursor of seminolipid. Additionally, KD can also be associated with the mutated activator protein, saposin A [32].

This disease damages the white matter of the brain; it is also known as globoid cell leukodystrophy. KD has been subdivided into four age/symptom-based types. The most common subtype, which presents faster progression, is early infantile type (birth–5 months) the symptoms include irritability, regression of psychomotor development, feeding difficulties, and, as the disease progresses, hypertonicity, seizures, loss of vision and hearing, and early death. At the other end of the spectrum is the adult type (>16 years) with slow development of progressive spastic paraparesis or gait abnormalities [33,34].

### 1.6. GM1-Gangliosidosis and Morquio B Syndrome

The deficient activity of the lysosomal hydrolase β-galactosidase (β-gal), coded by *GLB1*, can result in two distinguishable clinical entities, G_M1_-gangliosidosis and Morquio B syndrome. G_M1_-gangliosidosis is caused by lysosomal accumulation of ganglioside G_M1_ and its derivative G_A1_.

G_M1_-gangliosidosis is a neurodegenerative disease with G_M1_-ganglioside accumulation impairing neuronal functioning, whereas Morquio B is a progressive condition that mainly affects the skeleton and has an accumulation of keratan sulfate, which is excreted in the urine.

G_M1_-gangliosidosis is characterized by progressive neurologic dysfunction, presenting different degrees of severity depending on the subtype, which is subdivided according to the age of onset into Type I (infantile), Type II (late infantile/juvenile), and Type III (adult) [35].

Allelic mutations of the GLB1 gene cause heterogeneous clinical phenotypes, such as G_M1_-gangliosidosis and Morquio B syndrome. An important structural model of the protein was created and is convenient for modelling the mutant variants in order to better understand the structural bases of the diseases [36]. The existence of intermediate phenotypes that are clinically difficult to distinguish demonstrates the need to further investigate the disease. The in silico analysis and three-dimensional modelling of mutated GLB1 proteins are most helpful, but still not sufficient to fully differentiate between the two entities or establish a genotype/phenotype correlation [37].

### 1.7. Current Treatments for Sphingolipidoses

Currently, the most common treatments for sphingolipidoses are enzyme replacement therapy (ERT), substrate reduction therapy (SRT), and pharmacological chaperones (PC). ERT is currently the most widely used treatment. However, ERT has a variety of limitations. One major difficulty is that the replacement enzymes are not capable of crossing the blood–brain barrier. Additionally, all of the aforementioned therapeutic approaches are transitory and require periodically administration of the therapeutic agent. Furthermore, in the case of ERT, there is potential for an immunological reaction to the replacement enzyme. SRT is capable of targeting more than one sphingolipidosis as it utilizes inhibitors to block glycosphingolipid synthesis. However, this method can lead to partial depletion of glycosphingolipids from the plasma membrane, which can result in severe side effects due to off-targeting of the inhibitors (even targeting nonlysosomal enzymes) or other unknown interactions. Unlike most ERTs, some of the molecules used for SRT are able to cross the blood–brain barrier. PC treatment can only be successfully used in variants of diseases that are caused by enzyme misfolding, as chaperones assist in protein folding processes. As in the case of STR, this method is also prone to off-targeting [4,5].

ERT was the first therapy applied to sphingolipidoses and it resulted from a series of observations from various scientists; in terms of therapeutic advances, it was most important and provided the stepping stone for many other ERTs that are now available [38]. Concomitantly, it also brought upon these rare diseases a great interest from social, scientific, medical, and pharmaceutical points-of-view.

## 2. CRISPR-Cas9 and Prime Editing Background

CRISPR-Cas9 (clustered regularly interspaced short palindromic repeats) is a programmable tool for site-specific gene editing with low cost, easy application, and high efficiency when compared to other well-known gene editing tools like TALEN (transcription activator-like effector nuclease) and ZFN (zinc finger nuclease [39].

Type II CRISPR-Cas9 complex, as the name suggest, is composed of the Cas9 nuclease and a guide RNA (gRNA). When bound to DNA correctly, the Cas9 catalyzes a double-strand break (DSB) at the target site. Depending of the organism of origin, the Cas9 endonuclease can be structurally and functionally different. The most widely used and tested Cas9 is the *Streptococcus pyogenes* Cas9 (SpCas9), which requires a PAM (protospacer-adjacent motif) sequence downstream and adjacent of the target site to properly bind to the DNA sequence, which will then cut. For SpCas9 the PAM sequence is NGG, where N is any DNA nucleotide and G is guanine. This sequence occurs at a high frequency in the genome, allowing CRISPR-Cas9 to target virtually any gene. HNH and RuvC are the nuclease domains of Cas9 that allow for the DSB of the DNA [40]. When one of the domains is mutated, preventing the Cas9 from making DSB, a nickaseCas9 (nCas9) is produced. nCas9 is used to nick DNA, or to decrease off-targeting using two gRNAs complexed with nCas9 for DSB [41].

If both domains are inactive, the Cas9 complex acts as an inhibitor, preventing transcription of a certain gene by binding to the DNA area that constitutes that gene, but without cutting. This type of Cas9 tool is commonly known as dead Cas9 (dCas9) [42].

**Figure 2:**
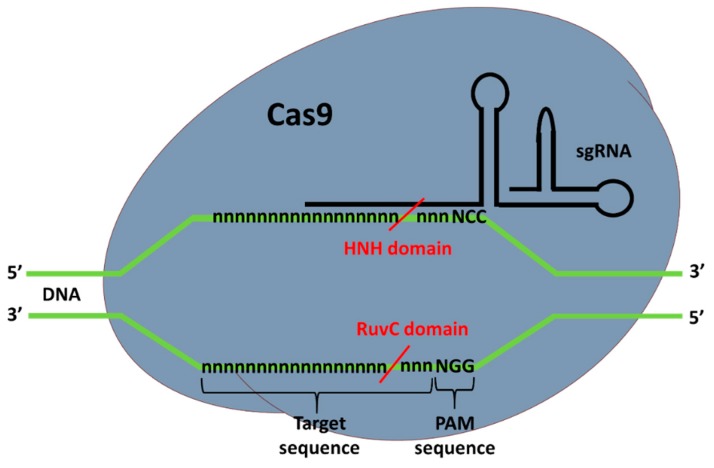
CRISPR-Cas9 structure and DNA binding for target sequence cleavage. “n” and “N” represent any nucleotide. The red lines indicate the cleavage locations made by Cas9 nuclease after CRISPR-Cas9 complex that binds to the target.

In addition to Cas9, a guide RNA is also necessary for targeting the DNA sequence. Together, CRISPR RNA (crRNA) and transactivating crRNA (tracrRNA) form the gRNA (two RNA chains bounded). This can also be fully synthesized in one piece, known as a synthetic single-guide RNA (sgRNA), composed of one RNA chain. gRNA binds to the Cas9 in order to form the ribonucleoproteic complex known as CRISPR-Cas9. The gRNA is complementary to the complementary chain of the target sequence and the crRNA part is composed of 20 nucleotides that bind to the DNA (Figure 2). The conserved 3′-end scaffold of gRNA (tracrRNA) binds to Cas9 due to the presence of a stem-loop structure in is end [40].

Even though there is the need for a PAM adjacent to the target site, the sequence (5′-NGG-3′) highly occurs in the genomes, thus, CRISPR-Cas9 can target virtually any gene. Even though this tool has many advantages, it also has its cons, a major one is the possibility of off-targeting due to homology with other sites [40].

The cleavage of the DNA creates a double-strand break (DSB) that is naturally repaired by the cell using one of two mechanisms. Usually it is repaired by the nonhomologous end joining (NHEJ) pathway, which introduces a small insertion or deletion (InDel) at the DSB site. However, the InDel normally disrupts the gene, which could subsequently impact structure and function of the resulting protein. The other pathway of repair, called homology-directed repair (HDR), uses, if existent (sister chromatid [43]) or added, a donor DNA fragment with homology to the flanking sequence that is integrated into the genome at the DSB site, thus repairing the broken DNA [44].

One of the limitations of using CRISPR-Cas9 technology for permanent gene modification is the efficiency of integration of the complex and donor template in living cells and the possible loss of cell viability due to the transfection process.

CRISPR-Cas9 integration can be carried out with plasmids or transcripts capable of expressing Cas9 and gRNA genes, or by directly transfecting the ribonucleoprotein (RNP) complex previously assembled in vitro [45].

The transformation can be achieved with electroporation, lipofection, polymeric nanoparticles, cell-penetrating peptides, or virus-like retroviruses and lentiviruses. However, disruption of the cell membrane is an aggressive procedure that results in many cell casualties. Furthermore, liposomes and viruses are limited by efficiency and size, and in vitro complexes can be toxic to the cells due to their exogenous nature [45].

Depending on the model and objective, the genome editing approach can change, but the highest nuclease activity is achieved with RNP. The RNP approach is reliable not only in cells, but also in embryos and in vitro assays. This approach is the most convenient and functional, since the complex synthesis is not dependent on DNA or mRNA expression by the cells to be edited. Moreover, it is the best method to avoid off-targeting due to the shorter exposure to the genome [46].

When a mutation in a specific gene leads to a nonfunctional protein, gene editing has the potential to repair this issue. In cases where a few mutations represent a large proportion of the causal alleles, it is possible to correct each mutation independently. Examples of such an approach exist in other pathologies, such as Duchenne muscular dystrophy [47], cystic fibrosis [48], chronic myeloid leukaemia [49], dominant progressive hearing loss [50], and autosomal dominant retinitis pigmentosa [51]. In brief, with CRISPR-Cas9, DSB can be performed in the mutated gene to correct the nonfunctional gene and potentially re-establish enzyme synthesis, and with an added donor DNA template (with a correct gene sequence), it is also possible to use DSB to insert a functional gene for synthesis of a functional enzyme.

Recently, a new revolutionary CRISPR-Cas9-based editing tool has been developed. This technology is known as Prime editing and can mediate targeted insertions, deletions, all 12 possible base-to-base conversions, and combinations in human cells without requiring DSBs or donor DNA templates.

This tool is composed of a nickase Cas9 fused with a reverse transcriptase (RT) that targets DNA using a guide RNA containing a spacer sequence that hybridizes with the target DNA. However, the sgRNA is engineered to have specificity to the DNA target and the new genetic information that replaces target DNA nucleotides. The DNA is nicked at the target site to expose a 3′-hydroxyl group that primes the reverse transcription of an edit-encoding extension on the engineered guide RNA (prime editing guide RNA—pegRNA) directly into the target site. This creates a heteroduplex with an edited and a nonedited strand. Subsequently, the cell’s natural repair mechanisms kicks in. To increase the chances of the DNA reparation mechanisms using the edited strand as template for reparation, a nick is created in the nonedited DNA. The nick is made with a normal sgRNA and nickase Cas9. This circumvents the heteroduplex issue by preferentially replacing the nonedited strand in the reparation process with a copy of the edited strand.

Prime editors1 (PE) use a RT fused to a RNA-programmable nickase and a pegRNA. PE2 uses an engineered RT to increase editing efficiencies, and PE3 nicks the nonedited strand to induce its replacement and further increase editing efficiency, typically to 20%–50% with 1%–10% indel formation (percentages of human embryonic kidney (HEK) 293T cell testing). PE3b is a PE3 system that additionally uses sgRNAs with spacers that match the edited strand, but not the original allele. This allows for a nick in the nonedited strand only after editing, minimizing concurrent nicks, DSB, and indel formation. Mismatches between the spacer and the unedited allele disfavor sgRNA nicking until editing of the PAM strand is completed. This system results in 13-fold lower average indels (0.74%) compared to PE3, without a decrease in edition efficiency [52].

## 3. CRISPR-Cas9 Edition for Disease Models and Therapeutic Approaches of Sphingolipidoses

Gene editing with CRISPR-Cas9 has been carried out to generate models of sphingolipidoses, and in a few such diseases, as an attempt at therapy (Table 1). In diseases where a partial reestablishment of function is enough to prevent the deleterious effect (such as many LSDs), CRISPR-Cas9 could be more widely used.

With CRISPR-Cas9, DSB can be performed in the mutated gene to correct the nonfunctional gene and potentially re-establish enzyme synthesis. With an added donor DNA template with a correct gene sequence, it is also possible to use DSB in order to insert a functional gene for synthesis of a functional enzyme. The use of Prime editing is also an option for the correction of specific nucleotide mutations, without the need of a DNA donor template or DSB.

### 3.1. Gaucher Disease

CRISPR-Cas9 has not been used yet for gene therapy in GD, but models have been generated (Table 1). Specifically, HEK 293T cells and A549 (adenocarcinomic human alveolar basal epithelial) cells have been used for disease modelling by knocking out the *GBA1* gene, using CRISPR-Cas9. The study aimed at observing the importance of the GCase enzyme in endocytic trafficking of viruses and exocytic transport. The results suggest diminished infection in both knockout cell lines when infected with influenza virus [53].

In addition to cellular models, in vivo models have been created. Recently, two zebrafish models that exhibit glucocerebrosidase deficiency have been generated using CRISPR-Cas9. Knockout of the genes *GBA1* and *GBA2* has been carried out in wild-type zebrafish AB/TL strain embryos. Once reaching adulthood, the knockout models were outcrossed to ABTL WT zebrafish. *GBA1* zebrafish mutants were heterozygous and *GBA2* mutants were homozygous. Double mutant larvae were then generated by crossing both *GBA1* and *GBA2* mutants. These models were used to study glucosylsphingosine and glucosylated cholesterol level variations in the presence of glucocerebrosidase deficiency [54].

### 3.2. Fabry Disease

Several researchers have applied CRISPR-Cas9 to FD. In 2016, researchers used CRISPR-Cas9 for gene knockout of *GLA* (in exon 1) in HEK 293T cells to create in vitro drug screening models for FD. More specifically, they aimed to design a FD model to study enzyme replacement therapy (ERT) with recombinant human α-Gal-A (rhα-GLA) [55].

A similar study also employed CRISPR-Cas9 to create a FD cell model to evaluate the amenability to chaperone therapy. In this case, the target was exon 3 of the *GLA* gene in HEK 293T cells. Although the target site was specific, single-cell analysis revealed different *GLA* mutations triggered by CRISPR-Cas9, partially leading to a complete loss of enzymatic function. Therefore, this model was successful in acquiring a disrupted *GLA* gene, but the method was not very encouraging since multiple mutations were found for a supposedly specific cutting tool [56].

In addition to HEK 293T cells, human embryonic stem cells (hESCs) have also been used to create FD models. A *GLA* knockout was generated for further differentiation into cardiomyocytes and proteome study. The model was made in order to study autophagic dysfunction and exosome secretion in FD-associated hypertrophic cardiomyopathy [57].

Other cases of FD models have been generated with other cell lines, namely human immortalized podocyte model of FD [58] and a human endothelial cell line, both with GLA disruption using CRISPR-Cas9 tools [59].

CRISPR-Cas9 technology has also been used to generate in vivo models including the *GLA*-knockout rat. The rat model is completely deficient in α-Gal-A activity; accumulates the established GSL biomarkers, Gb3 and lyso-Gb3; and develops mechanical pain behavior. This model was used to study the cation channel transient receptor potential ankyrin 1 (TRPA1) as a potential target to treat the pain of FD patients. Using the TRPA1 antagonist HC-030031, the mechanical pain behavior in Fabry rats was reversed with success [60].

Although CRISPR-Cas9 was, in these cases, used for disease model generation and not for gene/cell therapy, this work in models provides important insight for precise gene targeting of *GLA*. This background information can be used in future gene therapy and integration of a nonmutated *GLA* gene. Additionally, a good example of off-targeting occurrence, even in the presence of a highly promising off-targeting score, has been reported. Off-targeting is more than a prevision, it is always a real possibility.

In addition to disease modelling, gene editing for restoration of enzyme activity has also been done using CRISPR-Cas9. In 2017, Sheng-Kai Chang and collaborators restored GLA enzyme activity in FD patients’ fibroblasts after deleting the *GLA* IVS4 + 919 G > A mutation using CRISPR-Cas9. This specific mutation seems to be related to the presentation of cardiac FD issues, with greater rate of incidence in the Taiwanese population. The mutation interferes with the normal RNA splicing, producing a truncated GLA protein with no enzyme activity. The therapy was performed with CRISPR-Cas9, using two sgRNAs for the mutation deletion in FD patient fibroblasts, and demonstrated the proof of principle as an increase in GLA activity and clearance of intracellular Gb3 was observed. This work showed clear evidence that CRISPR-Cas9 has the potential for gene therapy directed to genetic diseases [61].

### 3.3. Tay–Sachs and Sandhoff Diseases

Both diseases are caused by mutations of *HEXA* or/and *HEXB* genes. Michael Tropak et al. achieved the disruption of *HEXA* and *HEXB* genes with CRISPR-Cas9 in HEK 293 cell line. Ultimately, they aimed to modulate the disease with the intent of testing a lab-made chimeric enzyme with a single hybrid µ-subunit. This subunit had a α-subunit active site, a stable β-subunit interface, and unique areas in each subunit for GM2AP (GM2 activator protein) interaction [62].

Allende and collaborators successfully performed gene correction using CRISPR-Cas9 to treat SD in patients’ cells. Fibroblasts of infantile SD patients were reprogramed to generate iPS cells that would be used for modelling the disease. The reprograming of diseased fibroblasts was first carried out via transfection of episomal vectors encoding the four reprogramming factors, OCT-3/4, SOX2, KLF4, and L-MYC. The resulting iPSCs had normal karyotype, pluripotent markers, and were able to form embryoid bodies that differentiated into the three embryonic germ layers. SD iPSCs showed reduced β-hexosaminidase activity compared with control iPSCs, which were purchased. Once the SD iPSCs model was prepared and tested, gene correction was performed. In order to correct the intron 10 acceptor splice-site mutation of *HEXA* gene, a plasmid was designed to express the sgRNA and the Cas9 nuclease for DSB catalysis and selection with puromycin. For correction, a single-stranded oligodeoxynucleotide of 181 bp was used as donor template. The template had a G to A correction, a silent point mutation to disrupt the PAM sequence for no Cas9 recutting, and two silent point mutations to create a KpnI restriction enzyme site for screening. Edited and not edited SD IPSCs were used for cerebral organoid formation that would mimic the first trimester of neurodevelopment. The result was accumulation of GM2 ganglioside, higher cellular size, and proliferation only in the unedited iPSCs [63].

Recent work using Prime editing, a new technique based on CRISPR-Cas9, has allowed for the correction of the most common frequent causal mutation in the Ashkenazi Jewish population. The mutation is a 4 bp insertion in *HEXA* (*HEXA* 1278 + TATC). PE3 prime editing was used to recreate the mutation by installation of the 4 bp insertion into *HEXA* gene of lipoinfected HEK 293T cell lines, resulting in 31% efficiency and 0.8% indels. Two isolated edited cells homozygous for *HEXA* 1278 + TATC mutation were then used to test 43 pegRNAs and three nicking sgRNAs with PE3 or PE3b systems for correction of the pathogenic insertion. Nineteen of the pegRNAs resulted in ≥20% successful editing reaching the best pegRNA, 33% efficiency, and 0.32% indels using PE3b [52].

### 3.4. Niemann–Pick Disease

In any disease, a cellular model is necessary to carry out gene and drug therapy tests. Although NPD type C1 is not exactly a sphingolipidosis, but rather a lipid transport disease, CRISPR-Cas9 applications in this pathology are worth mentioning. Many developments have been made in disease modelling. Descriptions of these developments are present in multiple types of sources, including articles and protocol books. One example is “Cholesterol Homeostasis: Methods and Protocols, Methods in Molecular Biology”, which describes with precision the generation of cellular NPC cholesterol storage phenotypes in *HeLa* cells using CRISPR-Cas9 to disrupt the *NPC1* gene [64].

Additionally, in vivo models, such as zebrafish NPC1-null mutants, have been generated using CRISPR-Cas9 gene targeting. Two models were made, one for early liver NPC1 disease and one for later neurological phenotype. sgRNAs, targeting exon 2 and 7, respectively, were injected with Cas9 mRNA into wild-type zebrafish embryos at the first cell stage. For mutation screening, the fish were raised to adulthood and outcrossed with wild-type adults for later PCR and fragment analysis [65]. A similar work has been also performed by Yusheng Lin et al. [66].

### 3.5. Krabbe Disease

In 2019, a work was published showing that *GALC*-expressing human neural stem cells (hNSCs) can be engineered to secrete lysosomal enzymes that are able to cross-correct Krabbe fibroblasts in vitro. To increase precision, multiple loci were targeted using Cas9 mRNA with modified synthetic gRNAs, along with DNA donor templates. The transplantation of the altered hNSCs into oligodendrocyte mutant shiverer-immunodeficient mice demonstrated migration and differentiation into astrocytes, neurons, and myelin-producing oligodendrocytes. They also generated altered hNSCs with *GALC* overexpression, capable of cross-correcting Galc enzyme activity via the mannose-6-phosphate receptor pathway. Since the *GALC* gene is overexpressed, it would be of great interest to use such cells in cell therapy for KD [67].

KD animal models have also been generated. For example, a gene knockdown of galactocerebrosidase in zebrafish was obtained in two *GALC* co-orthologs (Galca and Galcb) [68].

### 3.6. G_M1_-Gangliosidosis and Morquio Syndrome

Yvonne Latour et al. generated in 2019, a GM1-gangliosidosis model with purchased iPSCs, and consequently tested AAV9 gene therapy. Using CRISPR-Cas9, the researchers targeted exons 2 and 6 with different sgRNAs. The all-in-one plasmid method was used for expression of the complex in the cells after electroporation. The GLB1 knockout was confirmed by β-gal enzyme activity assays and DNA sequencing. The knockout iPSCs were then used to generate cerebral organoids that revealed progressive accumulation of GM1 ganglioside. Finally, AAVrh9 (AAV9) vectors carrying the human β-gal gene (AAV9-GLB1) or GFP (AAV9-GFP) were injected in GLB1 knockout organoids. The result was an increase in β-gal activity and concomitant reduction in GM1 ganglioside content in the AAV9-GLB1-injected organoids when compared with AAV9-GFP-injected organoids. This work contributed to the completion of the preclinical studies and progression to clinical trials using the AAV9-GLB1 vector as capable of reversing the phenotype [69].

Another recent work was able to generate GM1-gangliosidose and Morquio syndrome type B in mice models. Using CRISPR-Cas9, mutation W273L (position 274 in mice) was introduced into the GLB1 gene to generate a Morquio B model, while for GM1-gangliosidosis, a 20bp mutation was generated to remove the catalytic nucleophile of β-gal (β-gal-/-). The mice were generated by embryo microinjection of Cas9 protein, sgRNA targeting exon 8, and in the case of Morquio syndrome type B mice, a donor oligonucleotide to introduce a 2 bp mutation.

The generated Morquio syndrome type B mice showed reduction in β-gal enzyme activity, but no marked phenotype after one year. β-gal-/- mice lost β-gal enzyme activity, resulting in ganglioside accumulation and severe cellular vacuolation in the central nervous system (CNS), leading to neuromotor and neurocognitive dysfunction with disease progression. This was the first model of β-galactosidase deficiency with residual enzyme activity [70].

## 4. Conclusions

Since CRISPR-Cas9 was discovered, many advances have been made in gene editing for disease treatment that were previously impossible. The main targets of research are the most common and severe pathologies, such as cancer [71], which partly explains why there are still few gene therapies based in CRISPR-Cas9 for the rare sphingolipidoses [72,73].

CRISPR-Cas9 has been widely applied to generate knockouts for the study of the mechanisms underlying several diseases. The models created using CRISPR-Cas9 have already provided important insights. Additionally, this tool is being used to test the involvement of infection agents in the pathophysiology of diseases and the causality of novel genetic variants discovered through next-generation sequencing (Table 1 summarizes several applications of CRISPR-Cas9).

Commonly used therapies in sphingolipidoses have limitations, such as not being able to cross the blood–brain barrier or missing the target molecules. Some of these limitations can be circumvented by CRISPR-Cas9 genome editing. This tool has the potential to alter, with a high degree of precision, very specific sites of the genome. However, the off-target effects that arise from CRISPR-Cas9 editing cannot be disregarded as they can have deleterious effects. As with other techniques, multiple checkpoints are required at the gene and protein levels for the careful assessment of the results.

Undoubtedly, progress in the field of gene editing is rapid and further studies, not subjects of the present work, demonstrated that other approaches to gene therapy, such as RNA [74] or viral-based therapeutic approaches [75], provide useful insights for future applications. Prime editing already provides an improved tool for genome editing, based on Cas9, with the advantage of being less prone to off-targeting [52].

CRISPR-Cas9 has great potential for successful disease modelling, as well as for therapeutic approaches, avoiding problems inherent to other treatments. In light of the versatility of this technique, it is possible to envisage the development of other applications that have yet to be explored.

## Figures and Tables

**Table 1:** Sphingolipidoses and CRISPR-Cas9 use in disease models and gene/cell therapy.

Genes	Enzymes	Substrates	Disease	CRISPR-Cas9 for Disease Models:		CRISPR-Cas9 Gene/Cell Therapy	References
				Cell Models	*In vivo* Models		
GBA1	Glucocerebrosidase	Glucosylceramide	Gaucher disease	- HEK cells - A549 cells	- Zebrafish	X	[53,54]
GLA	α-galactosidase A	Globotriaosylceramide	Fabry disease	- HEK cells - hESCs - Podocytes - Human endothelialcells	- Rat - Mouse	- Restoration of GLA enzyme activity in FD patient′s Fibroblasts with mutation knockout	[55,56,57,58,59,60,61]
HEXA	α-hexosaminidase s-hexosaminidase	G_M2_-ganglioside	Tay-Sachs disease	- HEK cells	X	- Gene correction of HEXA 1278+TATC mutation in HEK293T cell using Prime editing	[62]
HEXB	α-hexosaminidase β-hexosaminidase	G_M2_-ganglioside	Sandhoff disease	- iPSCs	X	- No accumulation of GM2 ganglioside in iPSCs generated from fibroblasts of infantile SD patients due to template knockin	[52,63]
SMPD	Acid sphingomyelinase	Sphingomyelin	Niemann-pick diseases	- HeLa cells	- Zebrafish	X	[64,65,66]
GALC	β-galactocerebrosidase	Galactolipids	Krabbe disease	X	- Zebrafish	- Cell therapy with CRISPR-Cas9 edited hNSCs with correct enzyme activity	[67,68]
GLB1	β-galactosidase	G_M1_-ganglioside	G_M1_-gangliosidosis	- iPSCs	- Mouse	X	[69,70]
